# Identification of a Pro-Angiogenic Potential and Cellular Uptake Mechanism of a LMW Highly Sulfated Fraction of Fucoidan from *Ascophyllum nodosum*

**DOI:** 10.3390/md14100185

**Published:** 2016-10-17

**Authors:** Nicolas Marinval, Pierre Saboural, Oualid Haddad, Murielle Maire, Kevin Bassand, Frederic Geinguenaud, Nadia Djaker, Khadija Ben Akrout, Marc Lamy de la Chapelle, Romain Robert, Olivier Oudar, Erwan Guyot, Christelle Laguillier-Morizot, Angela Sutton, Cedric Chauvierre, Frederic Chaubet, Nathalie Charnaux, Hanna Hlawaty

**Affiliations:** 1 Inserm U1148, LVTS, Université Paris 13, Sorbonne Paris Cité, Paris 75018, France; nicolas.marinval@inserm.fr (N.M.); pierre.saboural@univ-paris13.fr (P.S.); haddad.oualid@univ-paris13.fr (O.H.); murielle.maire@univ-paris13.fr (M.M.); bassand.k@gmail.com (K.B.); robert.romain@gmail.com (R.R.); olivier.oudar@univ-paris13.fr (O.O.); erwan.guyot@aphp.fr (E.G.); christelle.laguillier@aphp.fr (C.L.-M.); angela.sutton@aphp.fr (A.S.); cedric.chauvierre@inserm.fr (C.C.); frederic.chaubet@univ-paris13.fr (F.C.); nathalie.charnaux@aphp.fr (N.C.); 2 Laboratoire CSPBAT, CNRS UMR 7244, UFR SMBH, Université Paris 13, Sorbonne Paris Cité, Bobigny F-93017, France; frederic.geinguenaud@univ-paris13.fr (F.G.); nadia.djaker@univ-paris13.fr (N.D.); khadijabenakrout@hotmail.fr (K.B.A.); marc.lamydelachapelle@univ-paris13.fr (M.L.d.l.C.); 3 Laboratoire de Biochimie, Hôpital Jean Verdier, Assistance Publique-Hôpitaux de Paris, Bondy 93140, France

**Keywords:** fucoidan, glycosaminoglycans, glycocalyx, angiogenesis, endocytosis

## Abstract

Herein we investigate the structure/function relationships of fucoidans from *Ascophyllum nodosum* to analyze their pro-angiogenic effect and cellular uptake in native and glycosaminoglycan-free (GAG-free) human endothelial cells (HUVECs). Fucoidans are marine sulfated polysaccharides, which act as glycosaminoglycans mimetics. We hypothesized that the size and sulfation rate of fucoidans influence their ability to induce pro-angiogenic processes independently of GAGs. We collected two fractions of fucoidans, Low and Medium Molecular Weight Fucoidan (LMWF and MMWF, respectively) by size exclusion chromatography and characterized their composition (sulfate, fucose and uronic acid) by colorimetric measurement and Raman and FT-IR spectroscopy. The high affinities of fractionated fucoidans to heparin binding proteins were confirmed by Surface Plasmon Resonance. We evidenced that LMWF has a higher pro-angiogenic (2D-angiogenesis on Matrigel) and pro-migratory (Boyden chamber) potential on HUVECs, compared to MMWF. Interestingly, in a GAG-free HUVECs model, LMWF kept a pro-angiogenic potential. Finally, to evaluate the association of LMWF-induced biological effects and its cellular uptake, we analyzed by confocal microscopy the GAGs involvement in the internalization of a fluorescent LMWF. The fluorescent LMWF was mainly internalized through HUVEC clathrin-dependent endocytosis in which GAGs were partially involved. In conclusion, a better characterization of the relationships between the fucoidan structure and its pro-angiogenic potential in GAG-free endothelial cells was required to identify an adapted fucoidan to enhance vascular repair in ischemia.

## 1. Introduction

Glycosaminoglycans (GAGs) are linear and sulfated carbohydrate chains covalently bound to a protein core to form a proteoglycan (PG), including syndecans [1]. The GAGs are shaped of sulfated disaccharide units composed of galactose or glucuronic/iduronic acid and *N*-acetyl-glucosamine/-galactosamine. As major components of the glycocalyx, GAGs, which cover the luminal outermost endothelial cell layer, are involved in angiogenesis, inflammation, as well as in cell proliferation, adhesion and migration [2,3]. Thus, reorganization of microenvironment, damages and modifications in the endothelial glycocalyx, caused by ischemia are widely studied [4]. Highly sulfated GAGs, such as heparan sulfate, mostly bind the signaling proteins (cytokines, chemokines and growth factors) and allow their retention/release, therefore contributing to glycocalyx and extracellular matrix reorganization [5]. It is known that the interaction of GAGs with signaling proteins involves the negative charges of the sulfates. However, we have previously shown that the relation between GAG expression and their potential in regulation of angiogenesis is difficult to characterize, mainly caused by the heterogeneity of their chain structure, sulfation level and position. Moreover, we also showed that the GAGs expression is subjected to modulation of expression pattern in size and sulfation levels during ischemia, modifying their ability to bind proteins [6].

Fucoidan, a marine sulfated polysaccharide from brown seaweeds that has similar biological activities of heparin, has been shown to promote revascularization in a rat critical hindlimb ischemia [7] and re-endothelialization in rabbit intimal hyperplasia [8]. Its polysaccharidic structure is mainly composed by fucose and uronic acid units, and confers to the fucoidan some properties which are similar in a certain extent to endogenous GAGs. It is noteworthy that this natural GAG mimetic could have comparable affinities for heparin binding proteins, such as chemokines and growth factors [9]. Depending of the type and size of polysaccharide fragments, the fucoidan could have a pro-angiogenic activity by modulating the bioavaibility of angiogenic cytokines in soluble or matrix-associated forms [10,11]. Recently, we demonstrated that the low molecular weight fucoidan (LMWF) modified the heparan sulfate expression pattern in modulating heparanase and syndecans expressions [12]. In addition, we have previously shown that the functionalized fucoidan present in three dimensional porous scaffolds was shown to retain the vascular endothelial growth factor (VEGF) and increased subcutaneous angiogenesis in mouse [13].

Upstream of developing a bio-engineering therapy based on fucoidan to regenerate damaged-vasculature, we propose the structure/function analysis to study its beneficial effect on angiogenesis and the endogenous GAG involvement in this process. Based on recent literature which showed the correlation between low molecular weight sulfated GAG-mimetics and their ability to regenerate damaged tissue [14], we hypothesized that the size and sulfation level of fucoidan could have an influence on cell migration and angiogenesis in glycocalyx-damaged human endothelial cells. We hypothesized that endogenous GAGs expression is altered in cardiovascular diseases and exogenous polysaccharides could modify the GAGs expression that we and others has already shown [12,15].

In our work we analyzed the correlation between the structure of the fucoidans and their functions on in vitro vascular network formation and endothelial cell migration in GAG-free human endothelial cells.

## 2. Results

### 2.1. LMWF and MMWF Fractions Collection and Characterization

#### 2.1.1. Fractionation and Composition of ASPHY, MMWF and LMWF

A column of size exclusion chromatography was used to elute the crude fucoidan Ascophyscient (ASPHY, 4100 g/mol) and collect two fractions with different molecular weight (Table 1), a medium molecular weight fucoidan (MMWF, 26,600 g/mol) and a low molecular weight fucoidan (LMWF, 4900 g/mol). Polydispersity analysis showed a very homogeneous population distribution of both polysaccharides LMWF and MMWF, as compared to the heterogeneous crude ASPHY (Table 1 and Figure S1). The composition of the three fucoidans (ASPHY, MMWF and LMWF) was analyzed then to determine sulfate, fucose and uronic acid mass percentage using the colorimetric measurement. The results showed the presence of fucose, sulfate and uronic acid in different percentage rate, 29%, 25%, and 27% for ASPHY, 36%, 29%, and 14% for MMWF and 21%, 23%, and 18% for LMWF, respectively (Table 2). The average density of sulfates for each fucoidan was calculated with a molecular rate of sulfate per fucose unit and showed that all fucoidans displayed a high sulfation rate (>1). The highest sulfation rate was attributed to LMWF (1.55), as compared to MMWF (1.14) and ASPHY (1.22) (Table 2).

#### 2.1.2. Raman and Fourier Transform Infrared Spectroscopy Analysis

**Figure 1:**
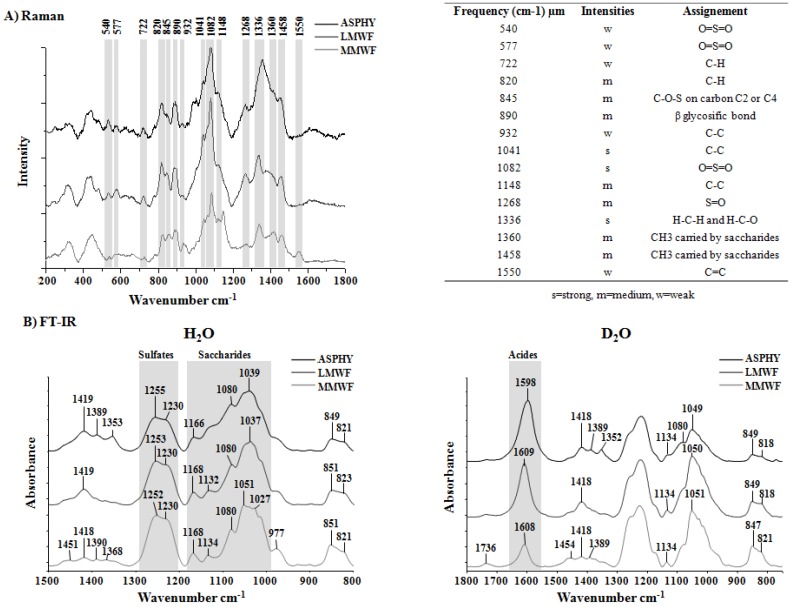
Raman and Fourrier Tansform Infrared (FT-IR) Spectroscopy analysis. Fucoidan spectra are represented in black for crude Ascophyscient (ASPHY), dark grey for the low molecular weight fucoidan (LMWF) and light grey for the medium molecular weight fucoidan (MMWF) for (**A**) Raman and (**B**) FT-IR (in H_2_O and in D_2_O). The numbers indicates the characteristics bands for polysaccharides.

Complementary to colorimetric measurement, the spectroscopic analysis of the three polysaccharides (ASPHY, MMWF and LMWF) was performed with Raman and Fourier Transform Infrared (FT-IR) Spectroscopy. The Raman band at 1458 cm^−1^ was assigned to scissoring vibration of CH_2_ and asymmetric bending vibration of CH_3_ for absorption at around 1455 cm^−1^, as suggested previously by Synytsya [16]. The Raman shoulder at 1360 cm^−1^ is originated from symmetric bending vibration of methyl and the FT-IR spectroscopy band at 1389 cm^−1^ could be the corresponding band already described at 1380 cm^−1^ (Figure 1A,B). The main pyranoid ring vibrations (HCC, HCO and COH) were observed in Raman band at 1336 cm^−1^, while COC stretching of glycosidic bonds and also CC and CO stretching covered the region located at 1200–900 cm^−1^. In Raman the β-glycosidic linkages between monosaccharide units was described at 890 cm^−1^. The characteristic band for sulfated polysaccharides attributed to asymmetric O=S=O stretching vibration (with some contribution of carbohydrate vibrations) was founded around 1253 cm^−1^ in FT-IR and 1268 cm^−1^ in Raman, although symmetric O=S=O stretching of sulfate was founded at 1082 cm^−1^ in Raman [17]. The Raman spectra of the three samples of fucoidans showed that LMWF exhibited a strong vibration at 1082 cm^−1^ and 1268 cm^−1^ compared to MMWF. For both LMWF and MMWF spectra, the Raman band at 845 cm^−1^ was attributed to COS bending vibration of sulfate substituents at the axial C2 and the equatorial C4 positions [18], both the 722 cm^−1^ and 820 cm^−1^ bands were attributed to the angular deformations of CH bonds. Otherwise the Raman band at 577 cm^−1^ and 540 cm^−1^ were attributed to the asymmetric and symmetric O=S=O deformation of sulfates [19]. The FT-IR analysis in D_2_O revealed the intensity of carboxylic groups (COO-) at the band 1609 cm^−1^ for LMWF and MMWF and 1598 cm^−1^ for ASPHY (Figure 1B). The data exhibited stronger intensities in the crude ASPHY and fractionated LMWF, as compared to MMWF.

In the next part of our work, in order to study the structure/function correlation of fucoidans, we analyzed the impact of ASPHY, MMWF and LMWF size and sulfation rate on human endothelial cell viability, angiogenesis and migration.

### 2.2. Biological Effects of LMWF and MMWF in GAG-Free Endothelial Cells

#### 2.2.1. LMWF and MMWF Affinities towards Heparin-Binding Proteins

**Figure 2:**
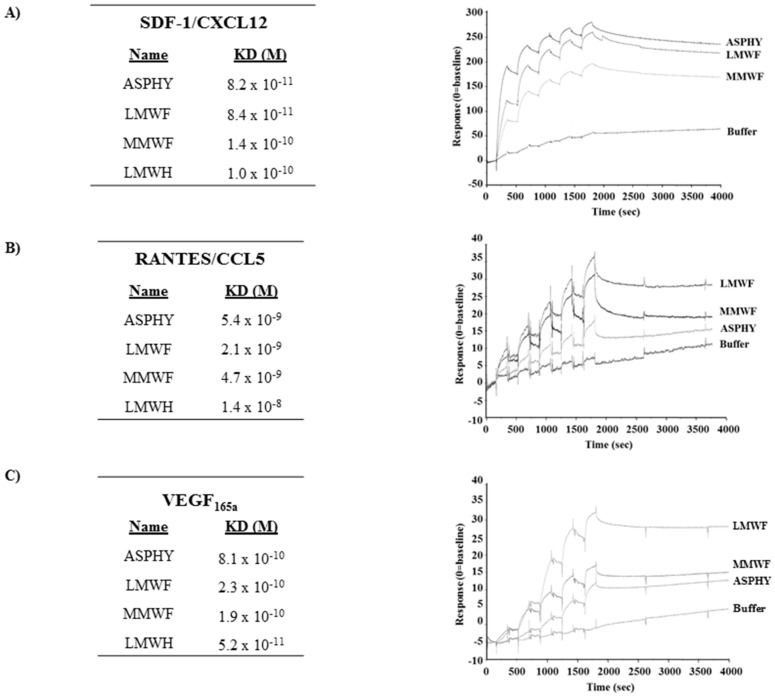
Affinity measurement of fucoidans to SDF-1/CXCL12, RANTES/CCL5 and VEGF. The binding responses of ASPHY, MMWF, LMWF and low molecular weight heparin (LMWH) to SDF-1/CXCL12, RANTES/CCL5 and VEGF were measured by Surface Plasmon Resonance. We immobilized biotinylated SDF-1/CXCL12, RANTES/CCL5 or VEGF on streptavidin chip. Each polysaccharide was injected over flow of a BIAcore sensor chip pre-coated with streptavidin biotinylated SDF-1/CXCL12, RANTES/CCL5 or VEGF. Each set of sensorgrams was obtained by injecting increasing concentration of polysaccharides (1.2, 3.7, 11.1, 33.3, and 100 nM). The response unit (RU) was recorded as a function of time (sec) and the affinities are expressed in molar (M) with the equilibrium dissociation constant KD (Kd/Ka). LMWH was used as a positive control of sulfated polysaccharide whereas non-sulfated dextran was used as a negative control (not shown). Affinity of polysaccharides to (**A**) SDF-1/CXCL12; (**B**) RANTES/CCL5 and (**C**) VEGF, and their corresponding representative sensorgrams.

We measured and compared the affinity of all the fucoidans towards the heparin-binding proteins (HBP) stromal derived factor-1 (SDF-1/CXCL12), regulated on activation, normal T cell expressed and secreted (RANTES/CCL5) and vascular endothelial growth factor (VEGF) by Surface Plasmon Resonance analysis. We used a low molecular weight heparin (LMWH) and a non-sulfated dextran (Dextran) as positive and negative control, respectively. Our data showed a characteristic model with a rapid association of the polysaccharide to the HBP and a slow dissociation as we have previously described [20] (Figure 2A–C). The results confirmed the direct interaction between fucoidans and HBP, characterized by an affinity KD (Kd/Ka), for SDF-1/CXCL12 (8.2 × 10^−11^ M for ASPHY, 1.4 × 10^−10^ M for MMWF and 8.4 × 10^−11^ M for LMWF) (Figure 2A), for RANTES/CCL5 (5.4 × 10^−9^ M for ASPHY, 4.7 × 10^−9^ M for MMWF and 2.1 × 10^−9^ M for LMWF) (Figure 2B) and for VEGF (8.1 × 10^−10^ for ASPHY, 2.3 × 10^−10^ for LMWF and 1.9 × 10^−10^ for MMWF) (Figure 2C).

There were no significant differences between the affinities of all fucoidans towards SDF-1/CXCL12 and RANTES/CCL5 and the affinities of LMWH (1.0 × 10^−10^ M, 1.4 × 10^−8^ M and 5.2 × 10^−11^ M respectively).

#### 2.2.2. LMWF and MMWF Effects on Endothelial Cell Viability

**Figure 3:**
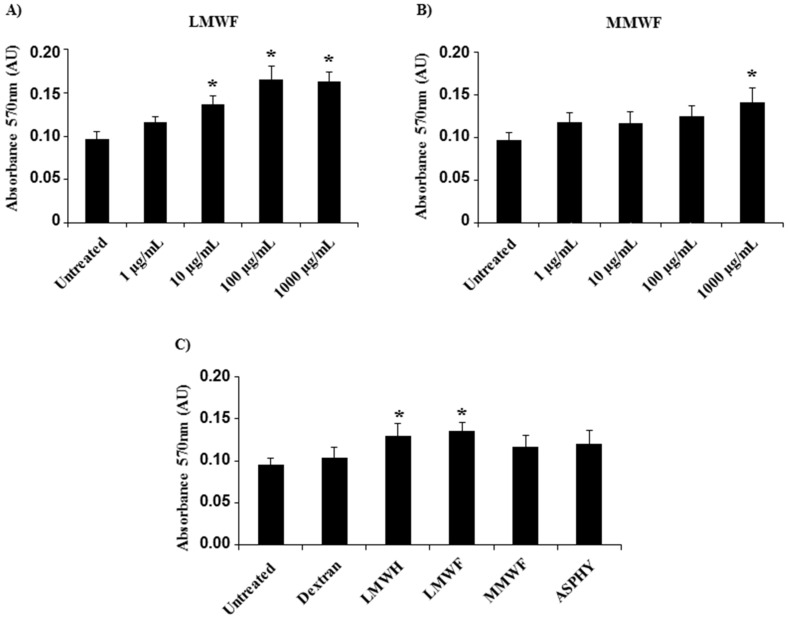
Effect of fucoidans on cell viability. The viability of HUVECs was analyzed by using MTT assay after fucoidan treatment for 24 h. The absorbance was read with a spectrophotometer (at 570 nm). HUVECs were incubated with (**A**) LMWF and (**B**) MMWF at increasing concentration (1, 10, 100, and 1000 μg/mL); (**C**) HUVECs were incubated 24 h with polysaccharides (dextran, LMWH, LMWF, MMWF and ASPHY) at 10 μg/mL. Values are expressed as means ± SEM (*n* ≥ 3). AU-Arbitrary units. * *p* < 0.05 versus Untreated.

We first studied the effects of fucoidans on human umbilical vein endothelial cells (HUVECs) viability using metabolic activity MTT test (Thiazolyl Blue Tetrazolium Bromide) after 24, 48, and 72 h of ASPHY, MMWF and LMWF treatments. Our results demonstrated that all fucoidans showed no toxicity for HUVECs from 1 to 1000 μg/mL, as compared to untreated cells (Figure 3 and data not shown). There was a light increase of cell viability after 24 h of LMWF treatment at 10, 100, and 1000 μg/mL (Figure 3A,C). However, only the highest concentration of MMWF at 1000 μg/mL increased the HUVEC viability (Figure 3B). At 10 μg/mL, LMWF increased the HUVEC viability (Figure 3C), but ASPHY and dextran did not have any effect on HUVEC viability at this concentration (Figure 2C and data not shown). In addition, there was no increase of HUVEC viability after 48 and 72 h of fucoidan treatment (data not shown). This assay established the viable culture conditions to measure angiogenesis and cell migration assays where we decided to use the fucoidans to analyze its biological activities at 10 μg/mL and up to 24 h of fucoidan treatment.

#### 2.2.3. LMWF and MMWF Effects on Angiogenesis In Vitro

**Figure 4:**
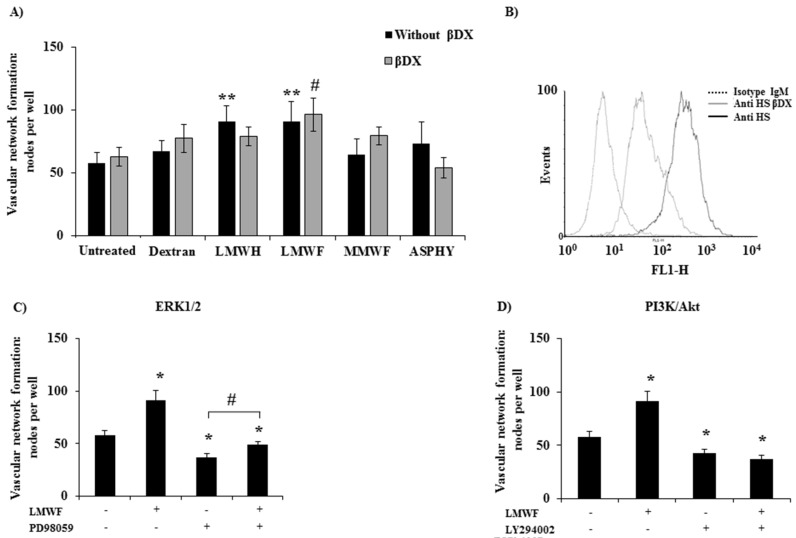
Pro-angiogenic potential of fucoidans on GAG-free HUVECs. (**A**) HUVECs pre-treated or not with βDX (4-Nitrophenyl-β-d-Xylopyranoside) were seeded on Matrigel and incubated with dextran, LMWH, LMWF, MMWF or ASPHY for 6 h. The cells were then stained with Hemalun Mayer’s and photographed for analysis. Values are expressed in number of nodes per well. ** *p* < 0.01 LMWF or LMWH versus Untreated (all without βDX). # *p* < 0.05 LMWF versus Untreated (all with βDX); (**B**) Endogenous GAGs expression analyzed by flow cytometry on HUVECs pre-treated or not 48 h with β*DX*; (**C**) PD98059, a pharmacological inhibitor of ERK1/2 and (**D**) LY294002, a pharmacological inhibitor of PI3K/AKT were added in HUVEC culture, then HUVECs were seeded on Matrigel for 6 h and vascular network formation was observed as described before. Values are expressed in nodes per well (*n* ≥ 3). * *p* < 0.05 LMWF, PD98059, LY294002, LMWF + PD98059, LY294002 + LMWF versus Untreated; # *p* < 0.05 LMWF + PD98059 versus PD98059.

In order to analyze the fucoidan structure/function relation in the angiogenesis processes, we established a 2-dimensional (2D) vascular network formation assay on Matrigel in vitro. The pro-angiogenic potential of ASPHY, MMWF, and LMWF at 10 μg/mL on HUVECs was analyzed as the percentage of cellular connection resulting in nodes formation per well at 6 h of incubation. Our results showed the significant increase of nodes formation by 56% ± 16% and by 57% ± 12%, after LMWF and LMWH treatments, respectively, as compared to control (Figure 4A, black bars). However, dextran, MMWF and ASPHY did not induce any changes in node formation (Figure 4A).

Recently, Kim et al. [21] reported that fucoidan from *Laminaria japonica* acts synergistically with fibroblast growth factor-2 (FGF-2) in promoting HUVEC angiogenesis by AKT signaling pathways via activation of the p38 and c-Jun N-terminal kinase (JNK) pathways. Based on these results, we performed the Western Blot analysis to verify whether MAPK/Erk1/2 or PI3K/AKT signaling pathways are implied in the pro-angiogenic effect of LMWF. To this aim, we incubated the cells with two pharmacological inhibitors PD98059 (for MAPK/Erk1/2) and LY294002 (for PI3K/AKT) before adding LMWF to cell culture. Our data attested that these two inhibitors significantly reduced the number of LMWF-induced nodes, by 46% ± 4.6% for PD98059 and by 59% ± 5.8% for LY294002, and evidenced the involvement of these signaling pathways in LMWF-induced angiogenesis from *Ascophyllum nodosum* (Figure 4C,D).

We have previously shown that fucoidan treatment can influence the syndecan-1/-4 and the glycosaminoglycan (GAG) expression level in HUVECs [12]. Since GAGs have been demonstrated to play an important role in angiogenesis processes, we studied the endogenous GAGs involvement in LMWF pro-angiogenic response. We established a GAG-free HUVEC model through the 4-nitrophenyl-β-d-Xylopyranoside (βDX) cell treatment for 48 h to inhibit GAG elongation. The efficiency of βDX on endogenous GAG abolition was verified by flow cytometry (Figure 4B). In these conditions, LMWF increased the vascular network formation by 53% ± 13%, whereas ASPHY, MMWF, LMWH and dextran had no effect (Figure 4A, grey bars). These results were similar with those obtained in basic condition with HUVECs expressing GAGs (56% ± 16%), demonstrating that the βDX treatment did not affect LMWF-induced angiogenesis.

These data suggests that endogenous GAGs were not involved in LMWF-induced angiogenesis, highlighting that LMWF had still a pro-angiogenic effect even in GAG-free condition.

#### 2.2.4. LMWF and MMWF Effects on HUVEC Migration In Vitro

**Figure 5:**
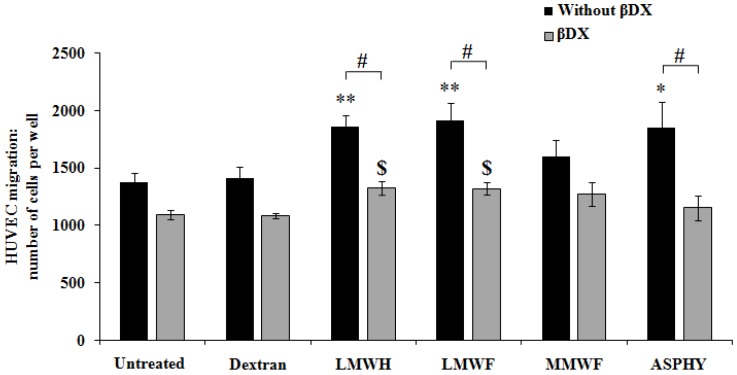
Pro-migratory potential of fucoidans on GAG-free HUVECs. HUVECs were seeded and incubated 24 h in the upper chamber with the polysaccharides dextran, LMWH, LMWF, MMWF or ASPHY at 10 μg/mL. The basal migration was performed in complete medium with 12% FBS. In the aim to remove the GAGs, the cells were pre-treated with βDX for 48 h, then the migration assay was performed with the same treatments as described above. The cells were fixed, stained with Mayer’s hemalun solution and counted after migration. Values are expressed as cell number per well. * *p* < 0.05 ASPHY versus Untreated (all without βDX); ** *p* < 0.01 LMWH and LMWF versus Untreated (all without βDX); $ *p* < 0.05 LMWH and LMWF versus Untreated (all with βDX); # *p* < 0.05 LMWH and LMWF and ASPHY (all without βDX) versus LMWH and LMWF and ASPHY (all with βDX).

To study the LMWF-migratory potential on HUVECs we assessed cell migration in a Boyden chamber. The HUVECs were incubated with ASPHY, MMWF or LMWF in the upper chamber (insert) and allowed to migrate through fibronectin-coated 8 μm-porous membrane to the lower chamber. The results showed a significant increase in HUVEC migration by 35% ± 16% for ASPHY, by 40% ± 11% for LMWF and by 36% ± 7% for LMWH, while MMWF and dextran did not have any effect on HUVEC migration (Figure 5).

Then we analyzed the involvement of endogenous GAGs in the pro-migratory effect of fucoidans. HUVECs were treated with βDX for 48 h and seeded in the upper chamber to obtain cell migration in GAG-free conditions. Our result showed that in this GAG-damaged condition, the effects of LMWF and LMWH on cell migration were significantly reduced by 31% ± 4% and 29% ± 4%, respectively. Both, LMWF and LMWH induced the migration in a light manner by 21% ± 5% and 22% ± 5%, respectively (Figure 5), suggesting that GAGs were partially involved in fucoidan-induced endothelial cell mobility.

In summary, LMWF was able to induce endothelial cell migration in Boyden chamber and this activity required the expression of endogenous GAGs to be fully effective.

### 2.3. Cellular Uptake of LMWF-Alexa in Endothelial Cells

#### 2.3.1. Regulation of LMWF Cell Uptake: Involvement of Endogenous GAGs

Very little is known about fucoidan localization and cellular uptake, which can be involved in HUVEC migration and vascular network formation. The fucoidan’s mechanism of action on the cells is still not well understood, however, heparin is known to be accumulated and internalized in endothelial cells by clathrin-mediated endocytosis [22] and it has been shown that the internalization pathway is related to the size of the polysaccharides [23].

**Figure 6:**
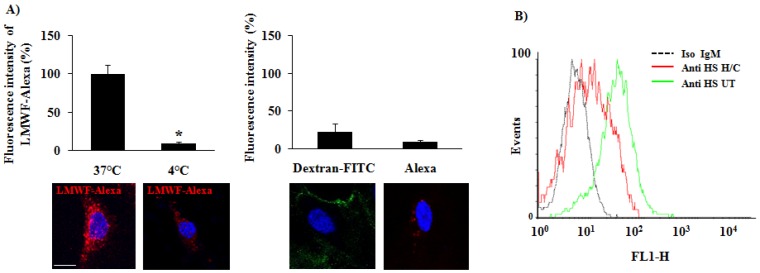

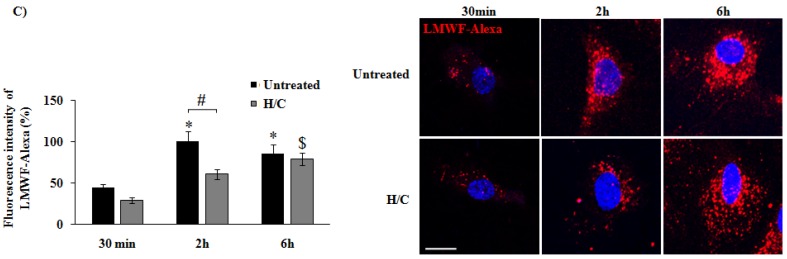
LMWF-Alexa localization in HUVECs by confocal microscopy. LMWF was previously coupled with the fluorophore Alexa Fluor 555. (**A**) LMWF-Alexa was added in HUVEC culture medium at 10 μg/mL during 2 h at 37 °C and 4 °C. Dextran-FITC and Alexa fluor alone (Alexa) were used as negative control. Pictures were taken by confocal microscopy and the intensity of the accumulated fluorescence per cell was quantified by using specific quantification software (DAPI—blue, LMWF-Alexa—red, Dextran-FITC—green, bar = 10 μm). Values are expressed as percentage of the intensity. * *p* < 0.01 37 °C versus 4 °C; (**B**) Endogenous GAGs expression on HUVECs treated by heparinase I, II, and III and chondroitinase ABC (H/C); (**C**) LMWF-Alexa was incubated with HUVECs (30 min, 2 h, and 6 h) with or without heparinase I, II, and III and chondroitinase ABC (H/C) and the intensity of fluorescence per cell was measured by flow cytometry. Values are expressed as percentage of the intensity normalized on the maximum intensity reached at 2 h. The right panel shows the confocal observation. (DAPI—blue, LMWF-Alexa—red, bar = 10 μm). * *p* < 0.05, 2 h, and 6 h versus 30 min (all Untreated with H/C); $ *p* < 0.05, 6 h versus 30 min (all treated with H/C); # *p* < 0.05, 2 h of treated with H/C versus 2 h of Untreated.

A fluorescent LMWF was designed by coupling the LMWF with a red fluorophore Alexa Fluor 555 (LMWF-Alexa). This technique allowed us to analyze the LMWF-Alexa cell uptake at physiological temperature (37 °C) and at low temperature (4 °C) that slow down the cell activity linked to membrane fluidity, dynamics and cell trafficking, thus reducing endocytosis. We measured the LMWF-Alexa accumulation in vesicle-like spots in HUVECs by the quantification of the red fluorescence intensity by confocal microscopy (Figure 6A). These vesicles-like spots reminded membrane movements similar to endocytosis. The control conditions were performed with Dextran-FITC (green fluorescence) and Alexa Fluor 555 (Alexa) alone (red fluorescence). The intracellular fluorescence was detected from 30 min of HUVEC incubation with LMWF-Alexa and reached the maximum of fluorescence intensity at 2 h of incubation at 37 °C. There was a very weak signal of fluorescence intensity detected for Dextran-FITC and Alexa alone at 2 h of incubation at 37 °C (Figure 6A, right panel). In addition, the LMWF-Alexa cell uptake was significantly decreased by 90% ± 2% at 2 h of incubation at low temperature at 4 °C (Figure 6A, left panel). These results evidenced the implication of the molecular chain structure of fucoidan (as compared to non-sulfated-dextran), membrane fluidity and dynamics in HUVEC uptake. The fluorescence intensity did not changed after 2 h of incubation (Figure 6C, black bars and data not shown) showing that the maximum of cell capacity of LMWF-Alexa uptake was reached at 2 h of incubation at 37 °C (Figure 6C, black bars).

We then used the HUVEC GAG-free model and tested the influence of endogenous GAG on LMWF-Alexa cell uptake up to 6 h. We performed an enzymatic degradation of the GAG using the heparinases I, III and chondroitinase ABC mix-solution (H/C) instead of long term culture with βDX treatment which was more appropriate for longer assays (from 6 h up to 24 h). The total endogenous GAG degradation was confirmed by flow cytometry (Figure 6B). Our results showed that the HUVEC pre-treated with H/C solution decreased significantly the fluorescence intensity by 40% ± 10% at 2 h of LMWF-Alexa incubation (Figure 6C, grey bars). Interestingly, H/C treatment (for GAG degradation) slow down the LMWF-Alexa cell uptake and the maximum fluorescence intensity was reached at 6 h of incubation at 37 °C. These results suggested that LMWF-Alexa cell uptake reached the saturable capacity of cells internalization starting from 2 h of incubation and the endogens GAGs are involved but not exclusive to regulate this process. In order to determine the mechanism of LMWF internalization in HUVECs we analyzed the different endocytic pathways.

#### 2.3.2. Mechanism of LMWF Uptake: Implication of Clathrin

**Figure 7:**
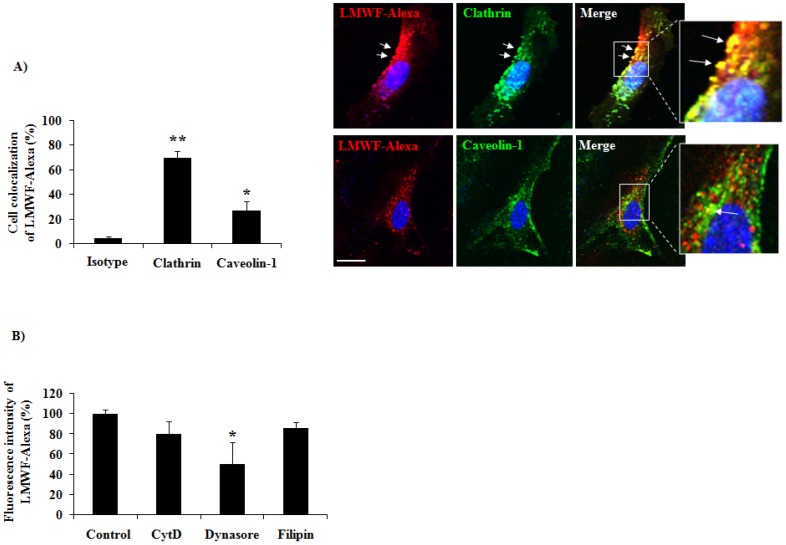
Internalization pathways of LMWF-Alexa in HUVECs analyzed by confocal microscopy. (**A**) HUVECs were incubated 2 h with LMWF-Alexa, fixed, permeabilized and the clathrin or caveolin-1 was revealed by immunofluorescence. A non-specific isotype of immunoglobulin was used as negative control (Isotype). The pictures were taken by confocal microscopy and the staining overlaped in merge (DAPI—blue, LMWF-Alexa—red, Clathrin—green, bar = 10 μm) high view inserts. The intensity of the fluorescence was quantified and the co-localization of markers was measured with the rate red/green represented in the histogram and dot plots. The intensity of fluorescence in HUVECs was quantified using specific software. * *p* < 0.05 Caveolin-1 versus Isotype; ** *p* < 0.01 Clathrin versus Isotype; (**B**) HUVECs were pre-treated or not (control) with specific inhibitors of endocytosis: Cytochalasin D (CytD-inhibits phagocytosis and micropynocytosis), Dynasore (inhibits clathrin mediated endocytosis) and Filipin (inhibits lipid raft formation) before adding LMWF-Alexa in the culture medium for 2 h. The intensity of fluorescence by cells was quantified as described above.* *p* < 0.05 Dynasore versus Control.

In this last part of our work we analyzed the mechanism of LMWF-Alexa endocytosis at 2 h of incubation using two specific markers of the receptor-dependent and receptor-independent endocytic pathways, clathrin and caveolin-1, respectively. We realized a co-localization assay of LMWF-Alexa (red fluorescence) with clathrin or caveolin-1 (green fluorescence) using confocal microscopy. A merge of the green and red fluorescent pictures were performed to see the co-localization of LMWF-Alexa with clathrin or caveolin-1. The fluorescence intensity per cell was quantified and the co-localization level was measured by scoring the proportion of red spots on green spots. The results showed that LMWF-Alexa was co-localized with clathrin at 70% ± 6% (Figure 7A, upper panel, arrows in the merge), but less co-localization was observed with caveolin-1 at 27% ± 7% (Figure 7A, lower panel, merge). This result suggested that LMWF-Alexa was mainly internalized by HUVECs in a clathrin-mediated endocytosis at 2 h of incubation.

To confirm this observation we used specific inhibitors of the major endocytic pathways: (a) the Cytochalasin D (CtyD), inhibitor of F-actin which acts on macropinocytosis/phagocytosis; (b) the dynasore inhibitor of the GTPase activity of dynamin which prevents the clathrin-coated pit formation and (c) the filipin which binds to cholesterol and inhibits the formation of lipid rafts and caveolin-vesicles. Our data demonstrated that at 2 h of incubation, the accumulation of LMWF-Alexa was decreased by 49% ± 6% in presence of dynasore, as compared to control (Figure 7B), however there was no changes of LMWF-Alexa cell uptake after CytD and filipin treatments. The transferrin was used as a positive control of clathrin-mediated endocytosis, and the results showed that the dynasore treatment decreased its cell uptake by 63% ± 4% (Figure S2).

In conclusion, our data evidenced that LMWF was internalized in HUVECs in a clathrin-dependant endocytosis.

## 3. Discussion

### 3.1. Structure-Function Correlation

For decades, natural marine sulfated fucanes named fucoidans, demonstrated anti-coagulant [24,25] and anti-thrombotic [9,26] effects comparable to heparin. We have previously shown that the fucoidans have therapeutic potential in cardio-vascular diseases in animal models with an important role in preventing intimal hyperplasia [8,27] and promoting revascularization after ischemia development [7]. We and other studies have shown the therapeutic effects of low molecular weight fucoidans (LMWF) on angiogenesis in vitro and in vivo [10,11,12,13,21,28].

Fucoidans are heterogeneous sulfated polysaccharides (size, composition and degree of sulfation), which can be obtained from different brown algaes, such as *Laminaria saccharina*, *L. digitata*, *Fucus evanescens*, *F. serratus*, *F. distichus*, *F. spiralis*, and *Ascophyllum nodosum*. Their structural diversity has been widely analyzed and described, highlighting an average structure based on a linear sulfated poly-fucose backbone with sometimes a few amount of uronic acids and traces of galactose and xylose. Many reports evidenced relationship between the structural features of fucoidans and their most potent biological activity, the widely admitted role of the molecular weight, and the sulfate groups content and distribution closely depending on the starting material and the method of preparation [9,25,29,30]. Thus, to conclude about structure-activity relationships could be tricky when considering different fucoidan fractions from the same origin. This is precisely well illustrated by works about the anti- and pro-angiogenic activity. Pomin et al. evidenced that fucoidans from various origins exhibit an anti-angiogenic activity due to their ability to interfere with Vascular endothelial growth factors (VEGFs) and basic Fibroblast growth factor (FGF-2) [30]. However, Matou et al. showed the pro-angiogenic effect of fucoidans, also extracted from *A. nodosum*, by enhancing the expression of α6, β1 integrin subunits and platelet endothelial cell adhesion molecule 1 (PECAM-1) on the surface of endothelial cells, resulting in an increase of FGF-2-induced angiogenesis [10]. Recently, Nifantiev et al. reviewed numerous studies on the angiogenic activities of fucoidans from different brown algae to highlight structure-activity relationships. They could only conclude that fucoidan fractions from *A. nodosum* with high molecular weight (>30,000 g/mol) exhibited anti-angiogenic activity whereas fucoidan fraction with low molecular weight (<30,000 g/mol) exhibited pro-angiogenic activity [31].

Furthermore, the heterogeneity in structure and composition of fucoidans appeared to correlate with heterogenous activities and the relation between the sulfate content and their anti-coagulant and anti-thrombotic potential was demonstrated by Ustyuzhanina et al. [32]. In this study, a native fucoidan with a degree of sulfation of 1.3 (*Saccharina latissima*) showed stronger activities than higher sulfated fucoidan from the same species, rather than other native fucoidans with a lower sulfation degree of 0.9 or 0.4 (*Fucus vesiculosus* or *Cladosiphon okamuranus*, respectively).

Only few publications presented the fucoidan effects on angiogenesis in endothelial cells or in cancer cells under hypoxic conditions. Chen et al. showed that a low molecular weight fucoidan (LMWF) obtained from *Sargassum hemiphyllum* (*M*_w_ = 760 g/mol, 40% sulfate) dose-dependly reduced hypoxia effect on VEGF-induced capillary tube-like structure formation in Human Umbilical Endothelial Cells (HUVEC) in vitro, and did not affect angiogenesis under normoxic condition [33]. In addition, they showed that LMWF treatment inhibited the migration and invasion of hypoxic Human Bladder Cancer Cells (T24). Interestingly, they suggested that under hypoxic conditions, the anti-angiogenic activity of LMWF in bladder cancer may be associated with suppressing Hypoxia-Induced Factor-1 (HIF-1)/VEGF-regulated signaling. Their experiments were performed in presence with both VEGF and fucoidan and it is well established that there are electrostatic interactions between negative charges from fucoidan and positive charges from VEGF, leading to depletion of both molecules that can explain their anti-angiogenic results. Cho et al. investigated the role of a fucoidan obtained from *Fucus vesiculosus* (*M*_w_ and composition non mentioned) [34]. They showed the decrease on Hepatocellular Carcinoma Cell (HCC) invasion in normoxic and hypoxic conditions and showed that fucoidan suppressed cells proliferation and invasion in a NDRG-1/CAP43-dependent manner. Teng et al. demonstrated that fucoidan from *Undaria pinnatifida sporophylls* (*M*_w_ = 104356 g/mol, 21% sulfate) significantly inhibits cell invasion and lymphatic metastasis in a mouse hepatocarcinoma Hca-F cell line under hypoxic conditions by suppressing HIF-1α/VEGF-C, which attenuates the PI3K/Akt/mTOR signaling pathways [35]. In conclusion, it is very difficult to compare these results with ours, since the experiments of Chen et al., Cho et al. and Teng et al. were performed with the fucoidans prepared from different seaweeds with different molecular weights, compositions and concentrations.

In this study, the characterization using Fourier Transform Infrared (FT-IR) and Raman Spectroscopy indicated that the crude ASPHY and the fractionated low and medium molecular weight fucoidans (LMWF and MMWF, respectively) demonstrated the characteristic bands of the O=S=O stretching vibration of sulfate, as determined through the observation of strong vibrational bands. The observation of the Raman band at 1268 cm^−1^ exhibited variations in intensity between the fucoidans with stronger band for LMWF. The Raman band of both LMWF and MMWF also exhibited vibrations at approximately 845 cm^−1^, which indicates the presence of sulfate groups at positions 2 and 4, respectively. Bilan et al. [36] obtained similar findings for the fucoidan extracted from *Fucus serratus Linnaeus*.

The FT-IR spectra showed that the intensity of COO- groups is higher in ASPHY and LMWF than in MMWF that can be compared to the amount of uronic acid measured in the fucoidans (27%, 18%, and 14%, respectively).

The spectroscopic data related to sulfate and carboxylic groups of the fucoidans can be correlated to the sulfation rate and uronic acid content obtained with colorimetric measures.

The negative charges carried by these native sulfated polysaccharides allows electrostatic interactions with numerous positively charged proteins, such heparin binding proteins (HBP), shown with stromal derived factor-1 (SDF-1/CXCL12) [37] and growth factors, such as FGF-2 [10,38]. These interactions were thought to induce HUVEC cell migration and lead to new vessels formation in animal models of ischemia [7,13]. In the current paper, we showed that crude (ASHPY) and fractionated fucoidans from *A. nodosum* (MMWF and LMWF) bound to HBP (SDF-1/CXCL12, regulated on activated T cell expressed and secreted RANTES/CCL5 and vascular endothelial growth factor VEGF) with a high affinity. However, our results highlighted a tendency in the affinity between the fractionated fucoidans both highly sulfated (1.55 and 1.14) and two of the HPB SDF-1/CXCL12 and RANTES/CCL5. LMWF presents a higher sulfation rate and relative higher affinity (KD) for both, compared with MMWF, which owns a weaker KD with both. ASPHY and LMWF behave similarly considering the interaction with HBP. This result is in accordance with the hypothesis of the modulation of the affinity by the sulfation rate, however the difference in sulfate degree for LMWF and MMWF was probably not enough significant to distinguish strong variations in the affinity to bind HBP. We hypothesized that the molecular model of interaction of fucoidans with the HBP fixed on the chip was polyvalent and related to the fast association and the slow dissociation phases in accordance with previous study with the GAG-mimetics [20]. The hypothesis was proposed that the amino acids present in the binding site of SDF-1/CXCL12 (BBXB) to heparin can be similar to those which interact with fucoidans. We suggest for the further study the use of adapted sensorgram designed to measure the interaction of fucoidan with the HBP and compete with heparin on the heparin binding-site to validate this model. Structural studies could also evidence that the size of fucoidan is also an important criterion to discriminate its affinity to HBP by using a range of fucoidan owning the same degree of sulfation.

In Boyden chamber migration assay, both LMWF and ASPHY increased the HUVEC migration. The structural analysis of fucoidan could explain the reason why the native fucoidan had a pro-migratory activity similar to LMWF. As shown on the polydispersity measurement, ASPHY contains LMWF populations in majority as its molecular mass was measured at 4100 g/mol. We analyzed distinct effects on HUVEC migration influenced by LMWF and MMWF. These data suggested that the size of LMWF was important, as a high sulfation rate, for the sulfated polysaccharide to have a pro-migratory activity on HUVECs.

The same results were observed in 2 dimensional (2D) vascular network formation on Matrigel with LMWF which shown higher potential to induce nodes formation than MMWF. However the native fucoidan ASPHY did not shown any activity in this assay, demonstrating that the fucoidan activity analyzed in HUVEC migration could act in a different pathway than 2D angiogenesis assay. We also revealed in this study that 2D-angiogenesis induced by LMWF involved PI3K/AKT and the MAPK Erk1/2 pathways, in line with recent study that evidenced the activation of P-38 and JNK pathways [21].

In our studies we evidenced the role of the sulfate groups content and molecular weight of fucoidan fractions on their angiogenic properties, and we proposed some preliminary mechanisms. This is possible because the fractions were obtained from a reliable and reproducible industrial process, that constitued a prerequisite for relevant studies. Anyway these fractions remain complex mixtures of macromolecules and determining a complete structure-activity is a difficult task that we had undertaken.

### 3.2. Influence of the Endogenous GAGs in Pro-Angiogenic Effect of Fucoidans in GAG-Free HUVEC Migration

Beside, in this study we explored the role of endogenous GAGs fucoidan-induced angiogenesis and HUVEC migration. We hypothesized that in basal condition, fucoidan could act as an intermediate actor between receptors and chemokines by increasing their concentration at the cell surface in co-operation with the endogenous GAGs. This interaction could enhance the formation of co-receptors (proteoglycans) and receptors complexes, their internalization and could lead to an amplification of the cell signal and response. While in absence of GAG expression, fucoidan could substitute the GAG function, prevent glycocalyx degradation and finally restore its function by enhancing the revascularization process. However, other hypothesis assumed that exogenous GAG-mimetics such as fucoidans, as they bind to HBP, could compete with endogenous GAGs for their binding sites leading to the inhibition of HBP activating pathways. Thus, suggesting that the pro-angiogenic activity of LMWF was not linked to its ability to bind HBP but mostly related to intrinsic activity.

We showed that LMWF still has a pro-migratory activity on HUVECs in GAG-free condition but in a lesser extent (40% ± 11% in basal condition versus 21% ± 5% in GAG-free condition). These results were not found for 2D-angiogenesis assay in GAG-free condition, where LMWF still induced nodes formation in the same range than basal condition. Endogenous GAGs shown in this field to be partially required for the fucoidan activity. This suggests that fucoidan could acts independently, depending on the biological effect. Otherwise, it has been proposed that fucoidan acts as a competitor for endogenous GAGs while binding HBP with higher affinity [11]. Considering the high affinity of LMWF to HBP, this model could partially correlate with our results as the tendencies showed that LMWF has stronger effect when the GAGs are expressed.

### 3.3. Internalization of LMWF

Our results attested that fluorescent LMWF was internalized in 2 h in HUVECs and localized in a perinuclear region. These observations correlated with a previous study focused on the heparin internalization in vascular smooth muscle cells, where the biphasic endocytic pathway of this sulfated polysaccharide was demonstrated [22].

The delay observed in the accumulation of LMWF-Alexa between untreated and Heparinase/Chondroitinase treated HUVECs demonstrated that endogenous GAGs were necessary to internalize the LMWF in 2 h. We evidenced that this internalization was temperature and clathrin-dependent. Proteoglycans are mobilized to induce clathrin-mediated endocytosis, and LMWF, as GAG-mimetic, could also interact with proteoglycan core or HBP at the cell surface and could be internalized and finally induced the biological effects on endothelial cells.

Previous studies have shown the importance of the size of polysaccharide in their cellular fluid internalization pathways with fluorescent low molecular weight dextran (10,000 g/mol) internalized in a clathrin and dynamin mediated micro- and macropinocytosis while medium molecular weight dextran (70,000 g/mol) used the amiloride-sensitive and clathrin-independent macropinocytosis [23]. Lately soluble exogenous GAGs have been demonstrated to improve cellular uptake of coated peptide-DNA complexes and escape from endosomal pathway until final localization in perinuclear region [39]. As suggested by our results, LMWF showed higher pro-angiogenic effect than MMWF. We then chose the LMWF to study its internalization and the potential implication of the GAGs on these mechanisms, which could explain the particular biological effects of LMWF on endothelial cells. Further trafficking studies should be proposed in correlation with cell signaling pathways to determine more accurately the cellular and molecular effects of LMWF on endothelial cells and its role inside the cells (signaling pathways) to induce angiogenesis.

## 4. Experimental Section

### 4.1. Reagents

Alexa Fluor Succinimidyl Ester (NHS Ester) was furnished by Molecular Probes (Thermo Fischer Scientific, Waltham, MA, USA). Transferrin-biotin labeled human (Sigma-Aldrich, Saint-Louis, MO, USA) was furnished by Sigma-Aldrich and used as a positive control of clathrin-mediated endocytosis.

### 4.2. Pharmacological Inhibitors

LY294002 (Sigma-Aldrich), is an inhibitor of the PI3K signaling pathway and was used at 30 μM in cell culture. PD98059 (Sigma-Aldrich) is an inhibitor of the MAPKK MEK1 and MEK2 signaling pathway and was used at 30 μM in cell culture. Cytochalasin D (C8273, Sigma-Aldrich) is an inhibitor of the phagocytosis and micropinocytosis by depolymerizing actin-F and was used at 100 μM in cell culture for 2 h. Filipin (F9765, Sigma-Aldrich) is an inhibitor of the clathrin independent endocytosis which binds to cholesterol and blocking membrane movements. This inhibitor was used at 1 μg/mL in cell culture for 1 h. Dynasore (D7693, Sigma-Aldrich) is an inhibitor of the clathrin mediated endocytosis by bocking the GTPase activity of dynamin and was used at 80 μM in cell culture for 30 min.

### 4.3. Enzymes and Glycosaminoglycan Substitute

βDX (4-Nitrophenyl β-d-xylopyranoside, Sigma-Aldrich), a substitute of glycosaminoglycans (GAGs) was used as specific inhibitor of the GAG chain elongation. βDX was added at 2 g/mol for 48 h in HUVEC culture to inhibit the endogenous GAGs expression before assays. Heparinase I (5U, 1/100) from *Flavobacterium heparinum* (H2519), Heparinase III (10U, 1/50) from *Flavobacterium heparinum* (H8891), and chondroïtinase ABC (10U, 1/50) from Proteus vulgaris were all obtained from Sigma-Aldrich and used 2 h at 37 °C to depolymerize endogenous GAGs in HUVEC culture before short time assays up to 6 h.

### 4.4. Antibodies

We used an antibody directed against heparan sulfate chains (Mouse IgM, Clone F58-10E4, AMS Biotechnology, Abingdon, UK) to observe their expression in flow cytometry CLTC is a primary antibody directed against clathrin heavy chain 1 (Mouse IgG1, Everest Biotech, Ramona, CA, USA). CAV-1 is a primary antibody directed against caveolin-1. PA5-17447 was obtained from Pierce (Rabbit IgG, Thermo Scientific, Rockford, IL, USA). The isotype Mouse IgG1 and the isotype Mouse IgM κ was obtained by BD Biosciences (BD Biosciences, Bedford, MA, USA), the isotype Rabbit IgG was produced by R&D (R&D Systems Inc., Minneapolis, MN, USA). The secondary antibodies Goat anti Mouse IgG Alexa fluor 488 was produced by Santa Cruz Biotechnology and Goat anti Rabbit IgG Alexa fluor 488 were produced by Santa Cruz Biotechnology (Santa Cruz Biotechnology, Santa Cruz, CA, USA).

### 4.5. Polysaccharides

The crude fucoidan (ASPHY) was obtained from the marine alga species *Ascophyllum nodosum* (Algues & Mer, Ascophyscient, batch #ASPHY12399, Ouessant, France). The Ascophyscient fucoidan was previously characterized by our laboratory [40]. A 10,000 g/mol non-sulfated dextran (Dextran T-10, Amersham Pharmacia Biotech, Amersham, UK) and a Dextran-FITC (TdB consultancy, Uppsala, Sweden) were chosen as negative controls. A low molecular weight heparin was used as a positive control (LMWH, *M*_w_ = 6300 g/mol, Tinzaparin sodium Innohep, Ballerup, Denmark).

### 4.6. Fractionation

The crude fucoidan ASPHY was fractionated using size exclusion gel. The column (XK 50/60, GE Life Sciences, Velizy-Villacoublay, France, id: 50 mm, L: 50 cm) was prepared with Bio-Gel P60 (Bio-Rad, Marne-la-Vallée, France). 0.15 M NaCl and 0.02% (*w*/*v*) sodium azide were used as carrier after careful filtration through 0.45 µm-filter unit (Millipore, Billerica, MA, USA) at 1.5 mL/min flow rate. 10 mg of Ascophyscient^®^ (30%, *w*/*v*) was eluted through the column and 100 mL were collected in several fractions. Then, the fractions were dialyzed five times against water (Spectra/Por, MWCO 1 kDa, Dominique Dutscher, Brumath, France) and freeze-dried. Each fraction was analyzed: fucose, uronic acid and sulfate rate were assessed by colorimetric assay and the molecular weight measured using size-exclusion chromatography with multi-angle laser light scattering-differential refractive index detection (HPSEC/MALLS-DRI) system [40]. We collected and used two fractions from the elution, a low molecular weight fucoidan (LMWF, *M*_w_ = 4,900 g/mol) and medium molecular weight fucoidan (MMWF, *M*_w_ = 26,600 g/mol).

### 4.7. Raman Spectroscopy

Raman spectra were recorded with a commercial confocal Raman microspectrometer from 200 to 1800 cm^−1^. A Horiba Scientific Xplora spectrometer was used at 660 nm excitation wavelength with 3 cm^−1^ spectral resolution. The Raman measurements were carried out in backscattering configuration through a 10× objective (NA = 0.25). The incident laser power was measured at the sample position and set at 20 mW. Raman spectra were acquired during 60 s acquisition time and baseline corrected using Labspec software (HJY, Kyoto, Japan).

### 4.8. Fourier Transform Infrared Spectroscopy

Fourier Transform Infrared Spectroscopy (FT-IR) spectra of the samples diluted either in D_2_O (>99.8% purity, Euriso-Top, CEA, Saclay, France) or in H_2_O solution were recorded on a Tensor 27 spectrophotometer (Bruker, Karlsruhe, Germany). Solutions were deposited between two ZnSe windows at a concentration of 333 μg/μL. Experiments were performed by drying the samples and dissolving them in solution at pH ~7. Twenty scans were accumulated (spectral region 4000–400 cm^−1^, resolution 1 cm^−1^). Data treatment was performed using the opus software and consisted of multiple point base line correction.

### 4.9. Surface Plasmon Resonance

Affinity of fucoidans for SDF-1/CXCL12, RANTES/CCL5 or VEGF_165_ was assessed with a BIAcore X100 (GE Healthcare, Freïburg, Germany). Biotinylated-SDF-1/CXCL12, -RANTES/CCL5 or VEGF_165_ was coupled to the surface of a SA sensor chip (carboxymethylated dextran with immobilized streptavidin for capture of biotinylated ligand). Biotinylated-SDF-1 (20 μL of 5 μg/mL in HEPES buffer saline-50 mM HEPES, pH 7.4, 150 mM NaCl, 3 mM EDTA, and 0.05% surfactant P-20) was then injected, at 5 μL/min flow rate, of the streptavidin-coated sensor chip to a resonance unit (RU) value of 110 for SDF1/CXCL12 and 235 for RANTES/CCL5. VEGF_165_ was immobilized on a CM5 sensorchip with the BIAcore amine kit at 1913 RU. Non coupled surfaces were used as controls, 1 M NaCl was used to regenerate the sensor surface. Samples were diluted in running buffer (50 mM HEPES pH 7.4, 150 mM NaCl, 3 mM EDTA, and 0.05% P-20). We selected with the BIAcore control software: temperature (25 °C), flow rate (30 μL/min), contact time (180 s), and sample volume (GE Healthcare) in single cycle method. Samples were injected consecutively at 1.2, 3.7, 11.1, 33.3, and 100 nM. The affinities of fucoidan for SDF-1/CXCL12, RANTES/CCL5 or VEGF_165_ were determined using a 1:1 Langmuir model by analysis the kinetic of the association and dissociation with the BIAcore evaluation software.

### 4.10. Endothelial Cell Culture

Human umbilical vascular endothelial cells (HUVECs, CRL-1730, ATCC, LGC Molsheim, France) were cultured in endothelial cell basal medium 2 (ECBM2, PromoCell, Heidelberg, Germany) supplemented with 12% fetal bovine serum, EGF (epidermal growth factor, 5.0 ng/mL), hydrocortisone (0.2 μg/mL), VEGF (vascular endothelial growth factor, 0.5 ng/mL), bFGF (basic fibroblast growth factor, 10 ng/mL), R3 IGF-1 (insulin like growth factor, 20 ng/mL), ascorbic acid (1 μg/mL) and antibiotics (penicillin-streptomycin, 1%, Invitrogen, Cergy, France) at 37 °C in 5% CO_2_. HUVECs were cultured at 60%–90% of confluence. The media were changed twice a week. The presence of growth factors such as VEGF, EGF, bFGF and IGF-1 in the culture medium of HUVECs, mimics the angiogenic conditions of in vitro cultures. We removed the heparin from the supplemented kit when the fucoidan treatment was performed, since heparin as a sulfated polysaccharide, could be considered as a competitor of fucoidan because of its structural and functional homologies.

### 4.11. Flow Cytometry

The level of heparan sulfate on HUVEC cell surface was quantified by fluorescence-activated cell sorting (FACS). Cells were pre-incubated for 1 h at 4 °C with anti-heparan sulfate antibody (10 μg/mL, Clone 10E4) or with isotypes (IgM). After washing, cells were labeled for 30 min at 4 °C with streptavidin-Alexa Fluor 488 complex (1/200, Molecular Probes, Invitrogen). Cells were fixed in 1% paraformaldehyde and analyzed with a FACScan (Becton Dickinson, Le Pont de-Claix, France).

### 4.12. Cell Viability Assay

The viability of HUVECs was demonstrated using MTT (3-[4.5-Dimethylthiazol-2-yl]-2.5-diphenyltetrazolium bromide) assay (Thiazolyl Blue Tetrazolium Bromide, Sigma-Aldrich). 5000 cells/well were incubated in a 96-wells plate for 24, 48, and 72 h with or without polysaccharides (LMWF, MMWF and ASPHY) at increasing concentration (1 μg/mL, 10 μg/mL, 100 μg/mL and 1000 μg/mL). LMWH and dextran were used as a control at 10 µg/mL. MTT solution at 1 mg/mL was added to the medium for 2 h and coloration was revealed in DMSO. The metabolic activity was correlated by the absorbance read at 570 nm with a photometer (Bio-rad^©^ Microplate reader, Model 680).

### 4.13. Cell Migration Assay

HUVEC migration was performed with Bio-coat cell migration chambers (Becton Dickinson, Franklin Lakes, NJ, USA) as described [26]. Briefly, inserts were coated with fibronectin (100 μg/mL, BD Biosciences). The polysaccharides (LMWF, MMWF, ASPHY, Dextran or LMWH) were added directly in the upper chamber at 10 μg/mL with 40 × 104 cell/wells in 500 μL of complete medium for 24 h. The lower chamber was filled with 500 μL of complet medium. The cytokines naturally contained in the complet medium were considered as basal inducer of chemotaxis migration. To test the role of endogenous GAGs, the cells were pre-incubated 48 h with βDX, and kept in inhibition condition for the experiment. Twenty-four hours later, medium and cells in the upper chamber were gently removed. Migrated cells in the lower chamber were fixed with methanol and stained with Mayer’s hemalun solution (Carl Roth GmbH + Co. KG, Karlsruhe, Germany). The cells were counted manually by two different observers who performed the blind data acquisition. HUVECs were photographed with phase contrast microscopy (Nikon^©^ Coolpix 8400, Nikon Corporation, Tokyo, Japan) at objective ×4 and quantified by using the Image J software (Rasband, W.S., ImageJ, U.S. National Institutes of Health, Bethesda, MD, USA).

### 4.14. 2D Vascular Network Formation Assay

The 2 dimensional (2D)-vascular network formation assay was performed with HUVECs cultured on Matrigel-coated (Corning, Bedford, MA, USA) 16-wells Labtek or 96 wells microplate. 1 × 10^4^ cells/well and incubated for 6 h at 37 °C in complete culture medium with or without 10 μg/mL of polysaccharides (LMWF, MMWF, ASPHY, Dextran or LMWH). The microvascular network was photographed using a phase contrast microscopy coupled camera (Nikon^©^ Coolpix 8400). The pictures were analyzed with 5 parameters: the total length network, the number of nodes (cell interactions), the number, the perimeter and the area of the generated meshes. The measures were estimated for each experimental condition using Image J analysis system. All measures showed same tendencies and we chose to exhibit the number of nodes that demonstrated higher differences between the treatments.

### 4.15. Labeling of the LMWF with a Fluorophore

LMWF was first aminated on the terminal aldehyde group of fucose chain by a reductive amination [41]. In this study, 50 mg of LMWF were solved in 0.54 mL solution of diaminopropan 1.5 M in glacial acetic acid and heated for 3 h at 90 °C, then a reduction was performed by adding 0.15 mL of dimethylboran 3 M in the solution and heated 3 h at 90 °C. Samples were dialized (cut-off 1000 Da) and freeze-dryed. LMWF was then coupled with a red fluorophore (Alexa Fluor^®^ 555 NHS Ester, Thermo Fisher scientific, Waltham, MA, USA). 1 mg of Alexa-Fluor 555 NHS was dissolved in 100 μL of dimethyl sulfoxide (DMSO, VWR BDH Prolabo, Fontenay-sous-Bois, France), beside, 10 mg of Aminated-LMWF was dissolved in 1 mL of carbonate buffer 0.1 M at pH 8.3 and 50 μL of the solution carrying the label was added to the solution stirred in darkness for 1 h at room temperature. The labeled compound was precipitated in ethanol to remove the free labels and eluted two times in column PD-10 (GE Healthcare Life Sciences).

### 4.16. Kinetic of the Cellular Localization of LMWF

Fluorescents LMWF-Alexa or Dextran-FITC (negative control) were added in HUVECs culture medium at 10 μg/mL in kinetic (30 min, 2 h and, 6 h) at 37 °C and 4 °C. The heparinases and chondroitinase were added to HUVECs culture. Cells were then fixed in paraformaldehyde 4%, stained with Dapi and observed under confocal microscopy (Leica SP8 tandem, Wetzlar, Germany) An average of 30 cells per condition was photographed in stack in the Z axe and the intensity of the accumulated fluorescence per cell was quantified by using specific quantification software (Imaris, Bitplane, Belfast, UK). The intensity gain was normalized on the auto fluorescence of the cells.

### 4.17. Co-Localization Assay of LMWF with Clathrin and Caveolin-1

HUVECs were incubated in presence of the fluorescent LMWF-Alexa for 2 h in the same conditions as described above, then fixed and permeabilized with saponine 0.1% (Fluka, Sigma-Aldrich). Cells were stained with Dapi and antibodies directed against light chain of clathrin (CLTC), caveolin-1 (CAV-1) or isotypes (Mouse IgG1; Rabbit IgG), then revealed by secondary antibodies (Goat anti mouse Alexa Fluor 488 and Goat anti goat Alexa Fluor 488) coupled with fluorophore. The colocalization assay was performed by using the specific quantification software Imaris.

### 4.18. Statistical Analysis

For the determination of statistical significance, an ANOVA test was performed with the Statview software (StatView 4.5 Abacus Concepts, Berkeley, USA). A *p* value of <0.05 was used as the criterion of statistical significance.

## 5. Conclusions

In summary, we showed in this study that from the heterogeneous crude fucoidan ASPHY collected from *A. nodosum*, we could distinguish two different fucoidans, LMWF and MMWF in their composition and through their pro-angiogenic effects. LMWF showed a higher activity to induce vascular network formation and endothelial cell migration than MMWF and ASPHY. These variations observed in their biological activities are mostly related to the size of the polysaccharide rather than their sulfation rate which were not enough different to distinguish significant variations in the affinity to chemokines SDF-1/CXCL12 and RANTES/CCL5. In addition, we demonstrated that the ability of LMWF to increase the vascular network formation at 6 h was regulated by Erk1/2 and PI3K/AKT cell signaling pathways. Localization study of LMWF-Alexa showed that the endothelial cells responded to fluorescent fucoidan presence by its uptake in a clathrin-mediated endocytosis and reached the maximum of cell capacity in 2 h with its accumulation in a perinuclear region.

Interestingly, we highlighted that the endogenous GAGs which were expressed at the surface of HUVECs, were partially involved in the pro-angiogenic activity of LMWF in vascular network formation and endothelial cell migration. LMWF activities were stronger when the endogenous GAGs were expressed, however in their absence LMWF has still an effect and showed that it could act as a substitute to induce angiogenesis and endothelial cell migration. The internalization of LMWF-Alexa was not inhibited but slowed down in GAG-free HUVECs. This work opens the way to use the most pro-angiogenic fucoidan as therapeutic GAGs substitute after glycocalyx injuries.

## Figures and Tables

**Table 1:** Molecular weight determination of fractionated fucoidans by HPSEC-MALLS-dRI.

Fucoidans	*M*n (g/mol)	*M*_w_ (g/mol)	Ip (*M*_w_/*M*n)
ASPHY	4100	10800	2.8 ± 0.6
MMWF	26,600	27,400	1.0 ± 1.2
LMWF	4900	5600	1.1 ± 1.2

**Table 2:** Composition of the fucoidans in fucose, sulfate, uronic acid and expression of the molar ratio sulfate/fucose.

Fucoidans	Fucose	Sulfate	Uronic Acid	Unknown	Ratio Sulfate/Fucose
ASPHY	29%	25%	27%	19%	1.22
MMWF	36%	29%	14%	21%	1.14
LMWF	21%	23%	18%	39%	1.55

## Supplementary Materials

The following are available online at www.mdpi.com/1660-3397/14/10/185/s1, Figure S1: Polydispersity analysis, Figure S2: Transferrin endocytosis.
