# Supplementary material for: Identification of a Pro-Angiogenic Potential and Cellular Uptake Mechanism of a LMW Highly Sulfated Fraction of Fucoidan from Ascophyllum nodosum
Source: Mar Drugs. 2016 Oct 17;14(10):185. doi: 10.3390/md14100185 (PMC5082333; doi:10.3390/md14100185)
Supplement: Supplementary file 1 [file marinedrugs-14-00185-s001.pdf]

Identification of a Pro-Angiogenic Potential and Cellular Uptake Mechanism of a LMW Highly Sulfated Fraction of Fucoidan from *Ascophyllum nodosum*

Nicolas Marinval, Pierre Saboural, Oualid Haddad, Murielle Maire, Kevin Bassand, Frederic Geinguenaud, Nadia Djaker, Khadija Ben Akrout, Marc Lamy de la Chapelle, Romain Robert, Olivier Oudar, Erwan Guyot, Christelle Laguillier-Morizot, Angela Sutton, Cedric Chauvierre, Frederic Chaubet, Nathalie Charnaux and Hanna Hlawaty

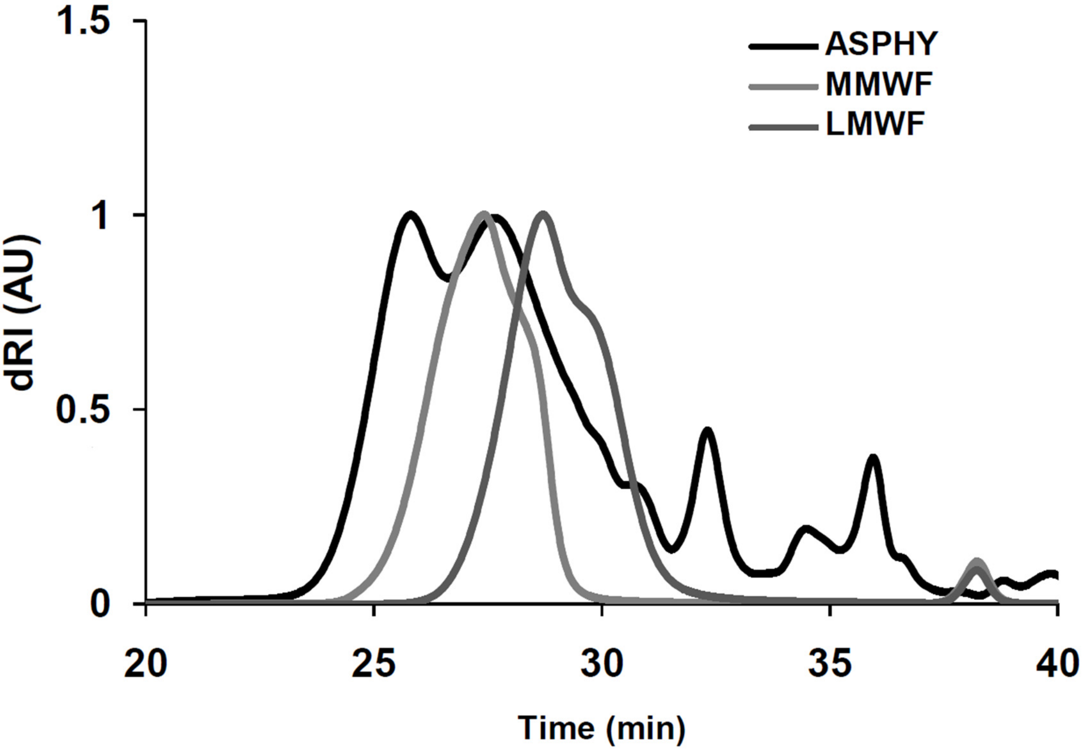

Figure S1. Polydispersity analysis. The polydispersity of the fucoidans ASPHY (**black**), MMWF (**dark grey**) and LMWF (**light grey**) represents the homogeneity of the population in this figure.

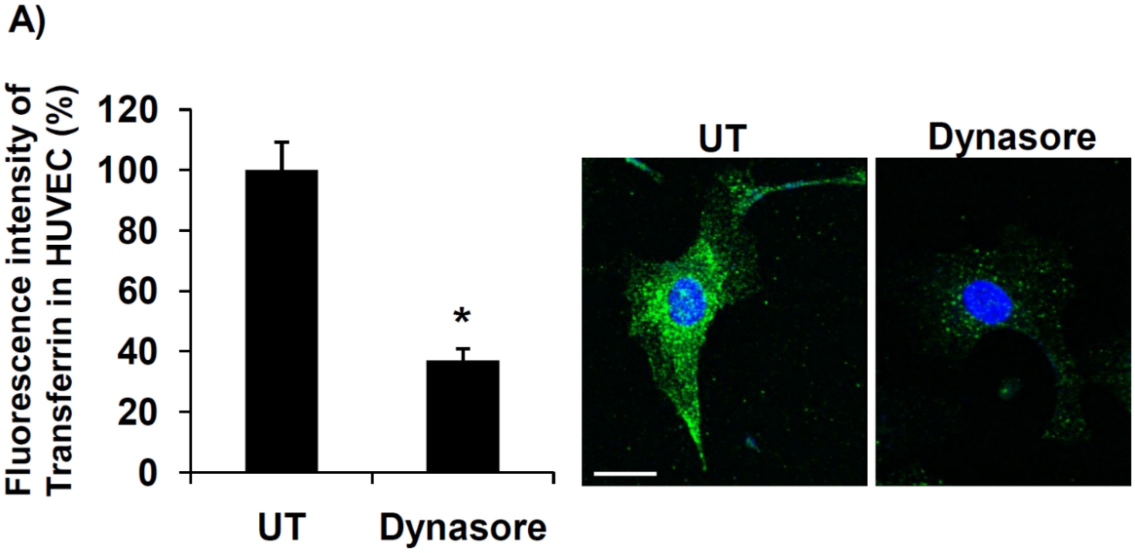

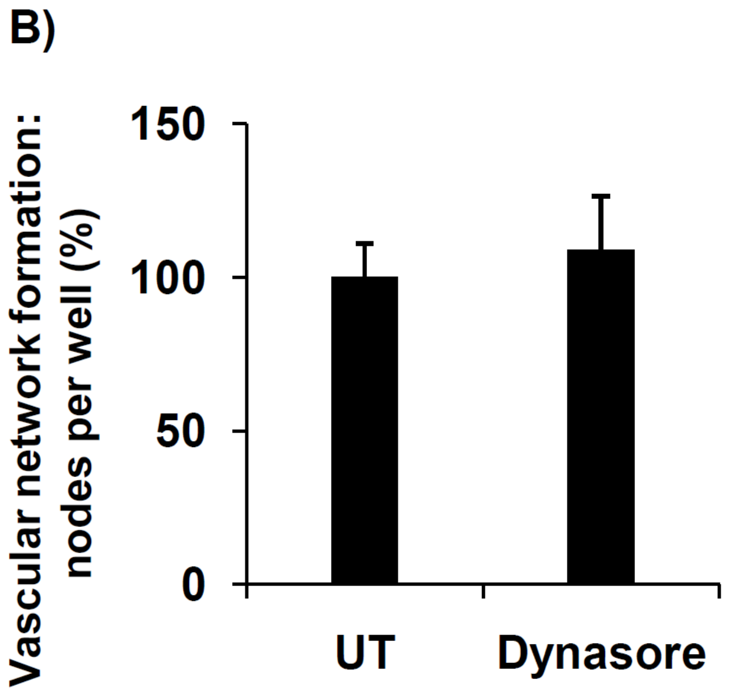

Figure S2. Transferrin endocytosis. (**A**) Transferrin has been used as a control of the inhibition of the clathrin-mediated endocytosis and its uptake in HUVECs was quantified after treatement or not (untreated—UT) with dynasore (Dapi—blue, Transferrin—green, bar = 10µm). * $p < 0.05$ Dynasore versus UT; (**B**) HUVECs were treated with dynasore 30 min before their seeding on Matrigel. The microvascular network formation was scored and presented as 100% of UT condition.
